# A Case of Amputation at the Shoulder Joint

**Published:** 1864-02

**Authors:** S. Barrett

**Affiliations:** Genesee County


ART. IV.—*A Case of Amputation at the Shoulder Joint, by* S. Barrett, M. D., *of Genesee County.*

On the 9th of December I received a telegram from Dr. Well, of Caledonia, requesting my immediate attendance at that place. On arriving there I found that G. B., aged 13, in attempting to get a ride on a freight train had fallen between the cars. The wheels had traversed his left arm to within about 3 inches of the shoulder joint, separating the bone and most of the flesh at that point, leaving the skin and thumb attached to it. The remainder was left on the track. On examination I decided at once to amputate at the shoulder joint. The necessary preparations having been made, he was brought fully under the influence of chloroform. The subclavian artery was compressed by my student, A. Dann, while Dr. Well steadied the stump of the arm by the skin that remained. I made a flap of the deltoid muscle in front, opened the capsule and carried the knife around the head of the bone, brought it down so as to make a second flap to correspond with the first. The axillary artery was secured, and but two others required ligating. He lost but little blood, although the operation was done in the night by the aid of lamps. There was a good deal of depression of the system from the shock of the injury, but he rallied under the influence of stimulants. The wound united with very little suppuration. He has made a good recovery and is now able to be out of doors.

